# The influence of hypoglycemia on the specific quality of life in type 2 diabetes mellitus: a comparative cross-sectional study of diabetics with and without hypoglycemia in Xi’an, China

**DOI:** 10.1186/s12955-021-01790-0

**Published:** 2021-05-19

**Authors:** Chao Wu, Yi-Ling Ge, Xin-Yan Zhang, Ming-Chao Liu, Chun-Ni Heng, Lin-Yuan Zhang, Yan-Ling Du, Shi-Zhe He, Lei Shang, Hong-Juan Lang

**Affiliations:** 1 Fourth Military Medical University, Xi’an, Shanxi China; 2 75Th Group Army Hospital, Kunming, Yunnan China; 3 Tang Du Hospital of Fourth Military Medical University, Xi’an, Shanxi China

**Keywords:** Type 2 diabetes mellitus, Hypoglycemia, Specific quality of life, Influence factor, China

## Abstract

**Purpose:**

This study aims to explore the incidence of hypoglycemia in patients with type 2 diabetes mellitus (T2DM) and the influence of hypoglycemia on the specific quality of life in T2DM patients.

**Methods:**

It was a comparative cross-sectional study consisting of 519 T2DM patients in Xi'an, China and patients were investigated by self-reported hypoglycemia and specific quality of life questionnaires from September 2019 to January 2020. Descriptive analysis, *t*-test, Chi-square test, hierarchical regression analysis and stepwise multiple regression analysis were applied to assess the influence of hypoglycemia on the specific quality of life.

**Results:**

The incidence of hypoglycemia in T2DM patients was 32.18%. The mean score of specific quality of life in diabetes without hypoglycemia was 57.33 ± 15.36 and was 61.56 ± 17.50 in those with hypoglycemia, which indicated that hypoglycemia had a serious impact on the quality of life of diabetics (*t* = − 5.172, *p* = 0.000). In the Univariate analysis of specific quality of life, age, education background, marital status, living status, duration of diabetes, monthly income per capita were independent and significant factors associated with specific quality of life of two groups of T2DM patients (*p* < 0.05). In the hierarchical regression analysis, the duration of the diabetes more than 11 years and the frequency of hypoglycemia more than 6 times in half a year entered the equation of specific quality of life of 519 diabetics respectively (*p* < 0.001). In multiple linear regression analysis, age, marital status and income all entered the regression equation of quality of life of the two groups (*p* < 0.05).

**Conclusion:**

Hypoglycemia will have a serious impact on the quality of life of T2DM patients. In order to improve the living quality in diabetics, effective measurements should be taken to strengthen the management of blood glucose and to avoid hypoglycemia.

## Introduction

As an increasingly common chronic disease, the incidence rate of Chinese diabetes is the highest in the world [1, 2]. Research data shows that from 1990 to 2016, the prevalence of diabetes in China increased from 3.7 to 6.6%, among which patients aged from 15 to 49 ranked the first [3]. A study of diabetes in China indicates that there were 56.4 million diabetic patients of working age in China, resulting in a loss of GDP as high as $2.6 trillion due to the decline of productivity in 2017 [4]. Diabetes not only causes physical damage to life span, but also has a serious impact on individual social interaction and psychological health, which impede social and economic development [5, 6].

Hypoglycemia is a common symptom and a relevant burden in T2DM patients [7]. Diabetics with hypoglycemia symptoms such as dizziness, nausea, physical fatigue, often due to the overdose of oral drug and injection of insulin therapy [8, 9]. Some studies have suggested that those diabetics with hypoglycemia were always in fear of potential hypoglycemia, significantly impairing their quality of life [10–12]. In recent years, more and more attention has been paid to the psychological health and the quality of life of T2DM patients [13–15]. Systematic review studies show that the prevalence of depression in diabetes is two or three times higher than that in patients without diabetes [16]. Diabetics with hypoglycemia are more likely to suffer from anxiety, depression and other psychological problems, which will affect the control of blood sugar and treatment effect [17]. As the country with the largest number of diabetic patients, China should not only underline the treatment of patients, but also the impact of the symptoms caused by diseases on their life. To further explore the effect of hypoglycemia on the quality of life of T2DM patients and its’ influencing factors in China, we adopt the method of comparative cross-sectional study and quantitative analysis so as to provide reference for the comprehensive prevention and treatment of diabetes.

## Methods

### Design

A comparative cross-sectional study and quantitative analysis of diabetics with data collection on the basis of questionnaires.

### Participants

Participants were T2DM patients from the Department of Endocrinology, Tangdu Hospital, located in the west of China. Participants who met the following criteria were eligible for the study: (1) patients with type 2 diabetes, (2) able to communicate in Chinese, (3) without hearing or reading impairment, and (4) voluntary participation in surveys. The exclusion criteria were patients with adrenal insufficiency or hypothyroidism that might cause hypoglycemia. The calculation of sample size is 5–10 times of the number of items in the scale, so at least 180 samples are needed in our study (N = (9 + 27) × 5 = 180). We explained the study design and the significance of research to the diabetics and reconfirmed that participants volunteered to participate in the survey and obtained their verbal and written informed consent. We adopted convenient sampling method to select participants and randomly sent invitations to 580 T2DM patients from September 2019 to January 2020. After the completion of the questionnaires, 519 valid questionnaires were finally confirmed.

### Questionnaire

The questionnaire consists of two parts: The first part is general demographic data. It was designed by the researchers, including 9 items such as gender, age, education background, frequency of hypoglycemia (if there is no hypoglycemia, participants do not need to answer). The second part is Diabetes Specific Quality of Life Scale [18]. This scale is different from the general quality of life scale. It is combined with the condition of diabetes to investigate the physiological conditions, psychological conditions, social relations and treatment of diabetes patients. It consists of 27 items and 4 dimensions via Likert's 5-level scoring method. Through the translation, back-translation, expert committee evaluation, pre-testing and psychometrics analysis of the Diabetes Specific Quality of Life Scale by Chinese scholar before our study, the transformation and cultural debugging of the scale were completed [19]. The scale has been adjusted by Chinese language and culture to be a Chinese version and is widely used in Chinese diabetes patients with good reliability and validity [20]. The higher the total score is, the more serious the damage of diabetes to the body and the worse the quality of life they will suffer. In this study, the Cronbach’s alpha coefficient was 0.853 and the test–retest reliability of the questionnaire was 0.968.

### Statistical analysis

Statistical analysis was conducted using a commercial statistical program (SPSS for Windows, version 23.0). According to the means of skewness and kurtosis, the scores of specific quality of life were normal distribution. The differences in characteristics between two groups were analyzed using the Chi-square test for categorical variables and the U of Mann–Whitney test was used to compare rank variables. The measurement data are described and counted in the form of mean ± standard deviation. The enumeration data are described and counted using frequency, percentage. T-test and variance analysis were used to compare between the groups in the univariate analysis of specific quality of life and its four dimensions and specific quality of life scores in T2DM patients. Hierarchical regression analysis of specific quality of life consists of three models. The first model was adjusted for gender, age, income, marital status, exercise, education level. In the second model, we additionally adjusted for duration of diabetes. Besides all the covariates in the second model, hypoglycemia was added in the third model. And the influencing factors of quality of life in the two groups were analyzed by multiple linear regression. All the tests were performed by two-sided test, with *p* < 0.05 as the statistical difference evaluation standard.

### Data availability

We used anonymous way to conduct questionnaire survey and the informed consent was signed by the participants. The research data are owned by the Department of nursing, Air Force Military Medical University which are not publicly available in order to protect personal privacy information of participants and the anonymity and confidentiality of the questionnaire results. The data are well managed and protected by the scientific secretary of nursing department. It can be obtained from the corresponding author (Email: 906963251@qq.com) upon reasonable request. Since the questionnaire was completed anonymously, if the participants wanted to know their research results, we would evaluate their questionnaire results on the spot and inform them of the specific quality of life scores.

## Results

### Characteristics of the participant

A total of 519 T2DM patients participated were included in this study, 352 of whom had no hypoglycemia, while 167 had hypoglycemia before. Of the 167 with hypoglycemia, 89 had hypoglycemia once or twice in half a year; 37 had hypoglycemia three to six times in half a year; 41 had hypoglycemia more than six times in half a year. Other general demographic data are shown in Table 1.

**Table 1:** General demographic data

Characteristics	Total sample	Diabetics without hypoglycemia		Diabetics with hypoglycemia		^2^/*U*	*P* value
	N	N	%	N	%		
*Sex*							
Male	329	228	69.30	101	30.70	0.900	0.343
Female	190	124	65.26	66	34.74		
*Age*							
≤ 20	8	6	75.00	2	25.00	− 1.777	0.076
21–40	108	75	69.44	33	30.56		
41–60	235	168	71.49	67	28.51		
≥ 61	168	103	61.31	65	38.69		
*Education background*							
Primary school or below	60	35	58.33	25	41.67	4.034	0.258
Junior high school	120	81	67.50	39	32.50		
Senior high school	254	173	68.11	81	31.89		
Bachelor degree or above	85	63	74.12	22	25.88		
*Marital status*							
Unmarried	49	34	69.39	15	30.61	3.074	0.215
Married	408	282	69.12	126	30.88		
Divorce or Bereavement	62	36	58.06	26	41.94		
*Living conditions*							
Live alone	84	53	63.10	31	36.90	3.090	0.378
Live with spouse	225	154	68.44	71	31.56		
Live with children	47	30	63.83	17	36.17		
Other	163	115	70.55	48	29.45		
*Duration of diabetes*							
≤ 3	157	115	73.25	42	26.75	− 2.622	0.009
4–5	68	50	73.53	18	26.47		
6–10	114	78	68.42	36	31.58		
≥ 11	180	109	60.56	71	39.44		
*Monthly income per capita in family*							
≤ 3000	210	136	64.76	74	35.24	1.766	0.413
3001–5000	146	100	68.49	46	31.51		
≥ 5001	163	116	71.17	47	28.83		
*Frequency of weekly exercise*							
No exercise	67	44	65.67	23	34.33	0.171	0.982
1–2 times / week	165	112	67.88	53	32.12		
3–5 times / week	101	69	68.32	32	31.68		
Exercise every day	186	127	68.28	59	31.72		
*Frequency of hypoglycemia*							
1–2 times/6 months	89			89			
3–6 times/6 months	37			37			
More than 6 time/6 months	41			41			

N, number; ^2^/*U*: Chi-square test/U-test

### Univariate analysis of specific quality of life and its four dimensions in T2DM patients with and without hypoglycemia

As shown in Table 2, for the group of diabetics without hypoglycemia, there were statistically significant differences in specific quality of life scores with different ages, education background, marital status, living conditions, duration of diabetes, income and exercise (*p* < 0.05). For the group of diabetics with hypoglycemia, the specific quality of life scores were statistically significant with different ages, education background, marital status, living conditions, duration of diabetes, income and frequency of hypoglycemia (*p* < 0.05).

**Table 2:** The Univariate analysis of specific quality of life and its four dimensions in T2DM patients

Characteristics	Diabetics without hypoglycemia					Diabetics with hypoglycemia				
	Specificity quality of life	Impact on physiology	Impact on psychology	Impact on social relations	Impact on treatment	Specificity quality of life	Impact on physiology	Impact on psychology	Impact on social relations	Impact on treatment
Sex										
Male	58.07 ± 14.29	24.67 ± 7.47	20.24 ± 5.13	7.33 ± 2.43	5.83 ± 1.95	61.83 ± 15.76	26.21 ± 8.13	21.58 ± 5.60	7.51 ± 2.48	6.52 ± 2.14
Female	55.96 ± 17.12	24.38 ± 8.80	19.03 ± 6.49	6.17 ± 2.04	5.84 ± 2.11	61.15 ± 20.00	26.91 ± 10.05	20.86 ± 7.50	6.83 ± 2.26	6.55 ± 2.37
*F* value	1.233	0.329	1.92	2.409	− 0.044	0.245	− 0.496	0.710	1.798	− 0.058
*P* value	0.219	0.743	0.056	0.017	0.965	0.807	0.621	0.479	0.074	0.953
Age										
≤ 20	47.50 ± 7.23	19.33 ± 4.37	17.50 ± 3.56	5.67 ± 1.37	5.00 ± 1.10	45.50 ± 2.12	18.00 ± 0.00	16.50 ± 2.12	5.00 ± 1.41	6.00 ± 1.41
21–40	48.44 ± 11.43	18.99 ± 5.28	17.28 ± 4.62	7.13 ± 2.44	5.04 ± 1.47	46.94 ± 12.53	18.00 ± 4.72	16.55 ± 5.19	7.30 ± 2.69	5.09 ± 1.57
41–60	57.34 ± 13.64	24.64 ± 6.95	19.85 ± 5.10	7.17 ± 2.23	5.68 ± 1.79	61.95 ± 14.69	26.78 ± 7.50	21.49 ± 5.52	7.31 ± 2.20	6.37 ± 1.89
≥ 61	64.35 ± 17.21	28.83 ± 8.63	21.74 ± 6.57	7.09 ± 2.41	6.70 ± 2.38	68.87 ± 17.78	30.63 ± 8.89	23.60 ± 6.57	7.22 ± 2.50	7.42 ± 2.44
*F* value	18.869	28.439	10.012	0.812	11.980	15.269	20.969	11.014	0.596	9.549
*P* value	< 0.001	< 0.001	< 0.001	0.488	< 0.001	< 0.001	< 0.001	< 0.001	0.618	< 0.001
Education background										
Primary school or below	67.71 ± 16.27	30.03 ± 8.53	23.06 ± 6.42	7.31 ± 2.19	7.31 ± 2.72	73.60 ± 14.31	32.72 ± 7.87	25.20 ± 5.94	7.20 ± 2.22	8.48 ± 2.28
Junior high school	61.26 ± 18.05	27.11 ± 8.65	20.49 ± 6.83	7.51 ± 2.63	6.15 ± 2.24	66.49 ± 21.34	29.26 ± 9.89	22.49 ± 7.97	7.85 ± 2.92	6.90 ± 2.48
Senior high school	55.14 ± 13.29	23.37 ± 7.04	19.28 ± 4.98	6.89 ± 2.26	5.60 ± 1.71	58.16 ± 14.80	24.73 ± 7.85	20.44 ± 5.43	6.99 ± 2.22	6.00 ± 1.88
Bachelor degree or above	52.51 ± 12.72	21.56 ± 6.73	18.62 ± 4.62	7.10 ± 2.11	5.24 ± 1.50	51.68 ± 12.84	20.95 ± 6.46	17.91 ± 4.60	7.18 ± 2.30	5.64 ± 1.47
*F* value	11.252	13.988	5.900	1.402	10.416	9.626	10.835	6.698	1.126	11.157
*P* value	< 0.001	< 0.001	0.001	0.242	< 0.001	< 0.001	< 0.001	< 0.001	0.340	< 0.001
Marital status										
Unmarried	51.41 ± 12.94	20.06 ± 5.94	18.26 ± 5.14	7.59 ± 3.02	5.50 ± 1.90	53.73 ± 14.40	20.33 ± 7.01	19.67 ± 5.79	7.73 ± 2.79	6.00 ± 1.604
Married	56.65 ± 14.71	24.34 ± 7.62	19.53 ± 5.37	7.05 ± 2.24	5.72 ± 1.93	60.40 ± 16.86	26.06 ± 8.51	20.73 ± 6.09	7.25 ± 2.38	6.37 ± 2.21
Divorce or bereavement	68.22 ± 17.64	30.58 ± 8.74	23.50 ± 6.95	7.11 ± 2.24	7.03 ± 2.35	71.73 ± 18.58	32.12 ± 9.04	25.00 ± 7.11	6.96 ± 2.39	7.65 ± 2.37
*F* value	12.643	17.366	9.697	0.806	7.572	6.582	9.848	5.620	0.484	4.228
*P* value	< 0.001	< 0.001	< 0.001	0.447	0.001	0.002	< 0.001	0.004	0.617	0.016
Living conditions										
Live alone	62.94 ± 20.24	26.79 ± 10.47	21.75 ± 7.21	8.00 ± 3.16	6.40 ± 2.58	68.71 ± 21.82	29.84 ± 11.41	23.84 ± 7.76	7.90 ± 2.88	7.13 ± 2.72
Live with spouse	56.19 ± 14.02	24.08 ± 7.16	19.36 ± 5.19	7.07 ± 2.20	5.68 ± 1.82	55.92 ± 15.22	25.24 ± 7.51	20.24 ± 5.63	7.25 ± 2.35	6.18 ± 2.05
Live with children	61.83 ± 16.05	27.70 ± 8.26	21.50 ± 6.16	6.30 ± 1.39	6.33 ± 2.37	66.35 ± 18.78	29.65 ± 9.65	23.24 ± 7.02	6.06 ± 1.35	7.41 ± 2.24
Other	55.09 ± 13.53	23.37 ± 7.20	19.10 ± 5.10	6.97 ± 2.11	5.65 ± 1.79	59.17 ± 15.95	25.04 ± 8.16	20.54 ± 5.89	7.23 ± 2.36	6.35 ± 2.05
*F* value	4.449	4.095	4.009	4.089	2.680	3.102	3.176	3.129	2.189	2.360
*P* value	0.004	0.007	0.008	0.007	0.047	0.028	0.026	0.027	0.091	0.073
Duration of diabetes										
≤ 3	51.39 ± 12.70	20.71 ± 6.35	18.36 ± 4.94	7.11 ± 2.50	5.21 ± 1.54	50.48 ± 13.42	20.50 ± 6.61	17.81 ± 5.68	6.88 ± 2.50	5.29 ± 1.38
4–5	56.80 ± 13.41	24.58 ± 6.76	19.50 ± 4.90	7.10 ± 2.30	5.62 ± 1.81	63.78 ± 15.05	26.78 ± 7.26	22.28 ± 5.06	7.89 ± 2.63	6.83 ± 2.07
6–10	57.95 ± 14.92	25.42 ± 7.54	19.78 ± 5.97	6.97 ± 2.18	5.79 ± 1.96	62.97 ± 17.34	27.86 ± 8.65	21.83 ± 6.71	6.81 ± 1.95	6.47 ± 2.46
≥ 11	63.39 ± 16.77	28.02 ± 8.55	21.54 ± 6.08	7.21 ± 2.26	6.61 ± 2.30	66.85 ± 17.65	29.52 ± 9.09	22.85 ± 6.29	7.52 ± 2.48	7.23 ± 2.28
*F* value	12.575	18.584	6.235	0.157	10.160	9.111	10.388	6.310	1.469	7.585
*P* value	< 0.001	< 0.001	< 0.001	0.925	< 0.001	< 0.001	< 0.001	< 0.001	0.225	< 0.001
Monthly income per capita in family										
≤ 3000	62.27 ± 17.20	26.82 ± 8.70	21.57 ± 6.33	7.43 ± 2.50	6.46 ± 2.44	67.42 ± 17.97	29.05 ± 9.08	23.45 ± 6.52	7.45 ± 2.45	7.47 ± 2.50
3001–5000	57.54 ± 15.36	24.78 ± 7.98	19.80 ± 5.56	7.26 ± 2.33	5.70 ± 1.76	63.78 ± 17.57	28.11 ± 9.02	21.78 ± 6.55	7.48 ± 2.47	6.41 ± 1.81
≥ 5001	51.34 ± 10.21	21.74 ± 5.94	17.78 ± 4.06	6.61 ± 2.01	5.22 ± 1.33	50.17 ± 9.94	20.85 ± 5.54	17.45 ± 3.98	6.70 ± 2.26	5.17 ± 1.24
*F* value	17.337	13.775	15.138	4.223	13.112	17.311	15.530	14.934	1.677	18.704
*P* value	< 0.001	< 0.001	< 0.001	0.015	< 0.001	< 0.001	< 0.001	< 0.001	0.190	< 0.001
Frequency of weekly exercise										
No exercise	54.14 ± 13.87	22.55 ± 7.06	19.45 ± 5.11	6.59 ± 2.38	5.55 ± 2.23	57.52 ± 16.00	24.30 ± 7.99	20.13 ± 6.01	6.61 ± 2.39	6.48 ± 2.45
1–2 times / week	59.70 ± 17.57	25.50 ± 9.08	20.67 ± 6.30	7.30 ± 2.46	6.22 ± 2.13	64.75 ± 20.15	27.94 ± 10.13	22.57 ± 7.12	7.32 ± 2.70	6.92 ± 2.33
3–5 times / week	59.86 ± 16.28	25.81 ± 8.13	20.75 ± 6.02	7.06 ± 2.45	6.23 ± 2.04	66.00 ± 18.76	29.09 ± 9.46	22.91 ± 6.75	6.84 ± 2.36	7.16 ± 2.30
Exercise every day	52.97 ± 12.60	23.77 ± 6.87	18.68 ± 4.85	7.15 ± 2.09	5.37 ± 1.68	57.86 ± 13.67	24.61 ± 7.24	19.75 ± 5.29	7.64 ± 2.13	5.86 ± 1.86
*F* value	3.203	2.481	3.312	1.018	5.044	2.635	2.802	2.868	1.396	3.280
*P* value	0.023	0.061	0.020	0.385	0.002	0.052	0.042	0.038	0.246	0.022
Frequency of hypoglycemia										
1–2 times/6 months						55.24 ± 14.83	23.39 ± 7.61	19.07 ± 5.58	7.04 ± 2.40	5.73 ± 1.62
3–6 times/6 months						68.41 ± 17.60	30.30 ± 9.07	23.62 ± 6.14	7.22 ± 2.39	7.27 ± 2.63
More than 6 times/6 months						69.12 ± 17.82	29.76 ± 9.10	24.05 ± 6.64	7.71 ± 2.44	7.61 ± 2.36
*F* value						14.492	13.177	13.337	1.064	14.629
*P* value						< 0.001	< 0.001	< 0.001	0.348	< 0.001

### Specific quality of life scores of diabetics with and without hypoglycemia

The scores of specific quality of life of the diabetics with hypoglycemia were higher than those without hypoglycemia. And the results of *t*-test in Table 3 showed that there were significant differences in total score (*t* = − 5.172, *P* < 0.001), dimension of impact on physiology (*t* = − 4.535, *P* < 0.001), dimension of impact on psychology (*t* = − 4.916, *P* < 0.001) and dimension of impact on treatment (*t* = − 6.523, *P* < 0.001) between the two groups.

**Table3:** Specific quality of life scores in T2DM patients

Items	Diabetics without hypoglycemia	Diabetics with hypoglycemia	*t* value	*P *value
Specificity quality of life	57.33 ± 15.36	61.56 ± 17.50	− 5.172	< 0.001
Impact on physiology	24.57 ± 7.95	26.49 ± 8.92	− 4.535	< 0.001
Impact on psychology	19.82 ± 5.67	21.30 ± 6.41	− 4.916	< 0.001
Impact on social relations	7.11 ± 2.32	7.25 ± 2.41	− 1.126	0.261
Impact on treatment	5.83 ± 2.01	6.53 ± 2.23	− 6.523	< 0.001

### Hierarchical regression analysis of specific quality of life of 519 T2DM patients

In Table 4, we assigned values to independent variables. In the hierarchical regression analysis of Table 5, the results showed that income, marital status, exercise, age, gender and education background, had entered the regression equation of quality of life of 519 T2DM patients (F = 27.090, R^2^ = 0.298, *P* < 0.001). On the basis of model 1, the duration of diabetes was included in model 2 (F = 21.397, R^2^ = 0.317, *P* < 0.001). Hypoglycemia was included in model 3 (F = 20.004, R^2^ = 0.322, *P* < 0.001).

**Table 4:** Assignment of independent variable

Independent variable	Assignment
Sex	Male = 1; Female = 2
Age	≤ 20 = 000; 21–40 = 001; 41–60 = 010; ≥ 61 = 100
Education background	Primary school or below = 000; Junior high school = 001; Senior high school = 010; Bachelor degree or above = 100
Marital status	Unmarried = 00; Married = 01; Divorce or bereavement = 10
Living conditions	Live alone = 000; Live with spouse = 001; Live with children = 010; Other = 100
Duration of diabetes	≤ 3 = 000; 4–5 = 001; 6–10 = 010; ≥ 11 = 100
Monthly income per capita in family	≤ 3000 = 00; 3001–5000 = 01; ≥ 5001 = 10
Frequency of weekly exercise	No exercise = 000; 1–2 times / week = 001; 3–5 times / week = 010; Exercise every day = 100
Frequency of hypoglycemia	1–2 times / 6 months = 00; 3–6 times / 6 months = 01; More than 6 times / 6 months = 10

**Table 5:** Hierarchical regression analysis of specific quality of life of T2DM patients

Independent variable	Specificity quality of life diabetics		
	M1	M2	M3
Monthly income per capita in family ≥ 5001 yuan	− 0.293***	− 0.296***	− 0.293***
Divorce or bereavement	0.139**	0.127**	0.125**
Exercise every day	− 0.133**	− 0.154***	− 0.153***
≥ 61 years old	0.430***	0.299***	0.300***
41–60 years old	0.319***	0.239***	0.244***
Monthly income per capita in family: 3000–5000 yuan	− 0.110**	− 0.108**	− 0.107*
Sex	− 0.125**	− 0.114**	− 0.116**
Education background: Senior high school	− 0.083*	− 0.092*	− 0.093*
Duration of diabetes 4–5 years		0.062	0.061
Duration of diabetes 6–10 years		0.082	0.078
Duration of diabetes ≥ 11 years		0.207***	0.198**
Hypoglycemia or not			− 0.070
*R*^*2*^	0.298	0.317	0.322
*ΔR*^*2*^		0.019	0.005
*F*	27.090***	21.397***	20.004***

*P < 0.05, **P < 0.01, ***P < 0.001

### Stepwise multiple linear regression analysis of specific quality of life of diabetics with and without hypoglycemia

For the group without hypoglycemia, the results of stepwise multiple linear regression analysis in Table 6 showed that gender, age, marital status, duration of diabetes, income, exercise were the influencing factors for the specific quality of life (F = 17.360, *P* = 0.000). For diabetics with hypoglycemia, age, marital status, income and frequency of hypoglycemia were the influencing factors for the specific quality of life (F = 19.420, *P* = 0.000).

**Table 6:** Stepwise multiple linear regression analysis of specific quality of life of T2DM patients

Personnel sorts	Independent variable	Specificity quality of life	Impact on physiology	Impact on psychology	Impact on social relations	Impact on treatment
Diabetics without hypoglycemia	Sex	− 0.119*		− 0.165**	− 0.154**	
	21–40 years old	− 0.221***	− 0.269***	− 0.165**		
	41–60 years old					0.210**
	≥ 61 years old		0.135*			0.304***
	Married					− 0.114*
	Divorce or bereavement	0.163**	0.127**	0.161**		
	Duration of diabetes ≥ 11 years	0.188***	0.154**	0.146***		0.189**
	Monthly income per capita in family: 3000–5000 yuan	− 0.120*		− 0.135*		− 0.130*
	Monthly income per capita in family ≥ 5001 yuan	− 0.291***	− 0.167***	− 0.288***	− 0.174**	− 0.218***
	Exercise once or twice a week		0.174**			
	Exercise three to five times a week		0.100*			
	Exercise every day	− 0.167**		− 0.186***		− 0.227***
*R*^*2*^		0.261	0.286	0.222	0.046	0.224
*F *value		17.360***	19.682***	13.994***	8.398***	14.179***
Diabetics with hypoglycemia	21–40 years old	− 0.337***	− 0.331***	− 0.303***		
	41–60 years old					0.261**
	≥ 61 years old		0.120			0.355***
	Junior high school				0.176*	
	Senior high school		− 0.128*			
	Married	− 0.143*		− 0.178**		
	Live with children				− 0.199*	
	Monthly income per capita in family: 3000–5000 yuan					− 0.219**
	Monthly income per capita in family ≥ 5001 yuan	− 0.272***	− 0.240***	− 0.246***		− 0.324***
	Exercise every day					− 0.159*
	Hypoglycemia 3–6 times / 6 months	0.208**	0.178**	0.203**		0.153*
	Hypoglycemia occurred more than 6 times / 6 months	0.263***	0.221**	0.260***		0.304***
*R*^*2*^		0.376	0.409	0.336	0.057	0.378
*F *value		19.420***	18.478***	16.290***	4.987**	13.825***

*P < 0.05, **P < 0.01, ***P < 0.001

## Discussion

As the world’s highest incidence of chronic non-communicable diseases, diabetes is the leading cause of major complications, such as end-stage renal disease and lower extremity amputations and is a significant contributor to ischemic heart disease, stroke and peripheral vascular disease [21]. The findings of our study revealed the poor overall living quality of T2DM patients in China, and the incidence of hypoglycemia in Chinese T2DM patients was 32.18% which was higher than the proportion of patients reporting hypoglycemic symptoms in Thailand (30.65%) [22] and lower than that in the United States (63%) [23]. There were 167 diabetics with hypoglycemia with the specific quality of life score being 61.56 ± 17.50 and 352 diabetics without hypoglycemia with the specific quality of life score being 57.33 ± 15.36, which both were in a relatively high score level.

Through statistical analysis, we found the living quality of those elderly, low education, low income, divorced or widowed, living alone, long duration of diabetes were worse and lower which was consistent with the findings of India and Nepal [24, 25]. Specific quality of life is an expansive concept that includes physical, social, economic, cultural and spiritual health, among others, which reflects the diabetics’ subjective perception and evaluation of his or her well-being and health, and it also can be influenced by multiple factors [26]. As the population ages and the chronic disease progresses, the number of elderly diabetics will continue to rise. Besides, due to the declines in body functions, vulnerabilities from comorbidities, psychological factors and geriatric syndromes such as frailty and cognitive impairment, the heavy multidimensional burden seriously diminished those elderly diabetics’ living quality [27]. Moreover, with the progression of the diabetes, the large physical, psychological and financial burden deriving from diabetes’ complications and treatment also increased. So the long duration of diabetes prominently diminished the specific quality of life [28]. Notably, it had been reported that patients with diabetes had lower levels of physical activity along with reduced overall fitness levels as compared to the general populations. Nevertheless, studies found that in those with diabetes, improvement in physical activity levels offers cardiometabolic, kidney, and cognitive benefits [27]. Exercise could enhance cardiovascular system function and physical feeling, improve insulin sensitivity, relieve blood pressure, blood lipids, and hence improve the effect of treatment. Therefore, nowadays, most expert guidelines have recommended or adopted a comprehensive chronic care model and precision diabetes medicine to optimize the diagnosis, prediction, prevention and treatment of diabetes by integrating multidimensional evaluation and multidisciplinary panel [29–31].

The results of t-test in Table 2 showed that compared with those without hypoglycemia, the score of specific quality of life in diabetes with hypoglycemia was higher significantly (*P* < 0.001), all in physiology, psychology, social relations and treatment. Besides, the variable of hypoglycemia was also included in the model 3 of hierarchical regression analysis in Table 5 which indicated that hypoglycemia could impaired the specific quality of life. Hypoglycemia is potentially one of the most severe acute adverse effects of therapies for diabetes. Elliott L [32] reported that the incidence rates of severe hypoglycemia in T2DM patients the incidence rates ranged from 0 to 20 per 100 person-years. Non-severe hypoglycemia events usually generate autonomic and neuroglycopenic symptoms which enable the individual to identify the onset, and to treat the falling blood glucose without requiring assistance while severe hypoglycemia is associated with impaired cognitive and physical functioning and the progressive neuroglycopenia interferes with the ability to self-treat [33]. Hypoglycemia and fear of hypoglycemia may further reduce adherence to glucose-lowering regimens, contributing to the further aggravation of diabetes-related complications. Therefore, it has a significant adverse impact on quality-of-life measures in diabetes [34, 35], which also supported our study’s finding. Psychologically, patients with recurrent hypoglycemia have been found to have chronic mood disorders including depression and anxiety; nocturnal hypoglycemia in particular may impact one’s sense of well-being on the following day because of its impact on sleep quantity and quality [27]. In the realistic scene, when diabetics have hypoglycemia, they will have a sense of near death in serious cases and if they are not treated in a short time, they will cause irreversible brain damage or even death, which scary them severely [36, 37]. Most of the time, the occurrence of hypoglycemia is not easy to detect, namely asymptomatic hypoglycemia [38]. Diabetics who have suffered from hypoglycemia will always worry that they will get hypoglycemia again [39].In the future blood glucose management, diabetics dare not make their blood glucose too low, causing a vicious circle of poor management of blood glucose and thus damaging their quality of life[22, 40, 41].

## Conclusions

With the development of aging society in China, the number and burden of diabetes are increasing [42]. Health China 2030 program proposes to improve the health level of the whole people [43]. Therefore, given that diabetes with such high incidence rate and being mortality worldwide, especially in China [44, 45] and hypoglycemia had such serious adverse effects on the diabetics’ living quality, effective approaches must been taken, particularly the measurements of decreasing the risk of iatrogenic hypoglycemia. On the one hand, medical staff should carry out health education on diabetes [46], which can learn from Karamanakos's [47] advocacy of community-based intervention for diabetes to carry out one-to-one guidance to strengthen blood glucose management and reduce the incidence of hypoglycemia. On the other hand, doctors should strengthen the screening of anxiety and depression in diabetics [48]. Studies found that early detection of anxiety and depression can significantly improve the quality of life of diabetics [49]. Besides, for patients, proper yoga and exercise [50, 51], increasing peer support and company can also alleviate depression, improve the quality of life of diabetics [52]. Diabetics can broaden their social contact by closely connecting with other patients and sharing their disease experience. In particularly for those with hypoglycemia, it is advisable to seek external help to deal with negative events and increase the level of resilience [53].

## Data Availability

The supporting data can be obtained by contacting the corresponding author by email (906963251@qq.com).
